# Post-mortem computed tomography in forensic shooting distance estimation: a porcine cadaver study

**DOI:** 10.1186/s13104-022-05997-2

**Published:** 2022-03-16

**Authors:** Juho-Antti Junno, Antti Kotiaho, Petteri Oura

**Affiliations:** 1 Cancer and Translational Medicine Research Unit, Faculty of Medicine, University of Oulu, Oulu, Finland; 2 Department of Archaeology, Faculty of Humanities, University of Oulu, Oulu, Finland; 3 Archaeology, Faculty of Arts, University of Helsinki, Helsinki, Finland; 4 Department of Diagnostic Radiology, Oulu University Hospital, Oulu, Finland; 5 Department of Forensic Medicine, Faculty of Medicine, University of Helsinki, P.O. Box 21, 00014 Helsinki, Finland; 6 Forensic Medicine Unit, Finnish Institute for Health and Welfare, Helsinki, Finland; 7 Center for Life Course Health Research, Faculty of Medicine, University of Oulu, Oulu, Finland

**Keywords:** Forensic medicine, Post-mortem imaging, Computed tomography, Gunshot wound

## Abstract

**Objectives:**

Gunshot wounds are frequently studied using computed tomography (CT) to examine tissue damage. In this study, we aimed to test the potential of post-mortem CT (PMCT) in shooting distance estimation at distances 0–100 cm. We hypothesized that in addition to the wound channel, we could also potentially detect tissue damage caused by muzzle pressure on PMCT.

**Results:**

A total of 59 gunshot wounds (23 contact shots, 21 close-range shots, 15 distant shots) were inflicted on eight piglet carcasses with a .22 Long Rifle handgun. PMCT scans were obtained using clinical equipment, and they were evaluated for wound characteristics by visual inspection and numeric measurements. In our data, contact shots could be clearly distinguished from close-range and distant shots by a hyperdense ring-shaped area surrounding the outermost part of the wound channel. Close-range and distant shot wounds did not have this feature and were difficult to distinguish from each other. The mean wound channel diameter ranged from 3.4 to 5.4 mm, being smallest in contact shots and largest in distant shots. These preliminary findings suggest that PMCT may aid the estimation of shooting distance. As this study only addressed low velocity gunshot wounds in carcasses, further studies are warranted.

**Supplementary Information:**

The online version contains supplementary material available at 10.1186/s13104-022-05997-2.

## Introduction

The vast majority of missile wounds are caused by firearms [1]. From the forensic pathologist’s point of view, the major details in gunshot-related incidents involve weapon type, bullet type, shot trajectory, and shooting distance. If there is lack of background information from the scene, these key details need to be inferred on the basis of wounds and injuries detected at autopsy. Although there are published guidelines to assist the forensic pathologist in interpreting gunshot injury by visual inspection [2, 3], other modalities such as post-mortem imaging may bring additional value to the process.

Gunshot wounds can be divided into two or three main categories: low velocity and high velocity gunshot wounds, 610 m/s being the dividing speed between these two categories. In three categories, the division is made at bullet velocities of 350 m/s and 610 m/s. In general, handguns perform at velocities below 610 m/s which is also the maximum for unjacketed bullets [4].

Mechanical trauma is generally characterized by the transfer of energy from an external object (e.g., a firearm) to the victim’s target tissues [1]. The wounding capability of a bullet is associated with its velocity and kinetic energy, the latter of which is also influenced by the weight of the bullet. Higher velocities increase the shock wave and the cavitation of tissue, in addition to direct damage caused by the passing bullet [5]. In general, high velocity bullets cause more severe bone and soft tissue damage even without a direct impact of the bullet, although serious injuries occur with low velocity bullets as well. In addition to velocity, other factors such as bullet type may play a major role in the efficiency of energy transfer and wounding capacity of the bullet [6].

The estimation of shooting distance is commonly based on measurable evidence, e.g., gunshot residue analyzed by means of an automated image analysis [7]. Although computed tomography (CT) is often used to evaluate gunshot wounds in the forensic context [8, 9], it is rarely utilized as a tool to estimate shooting distance [10].

In this study, we aimed to test the potential of post-mortem CT (PMCT) in shooting distance estimation at distances 0–100 cm. We compared PMCT scans of entrance wounds from three shooting distances. We hypothesized that in addition to bullet wound channel, we could also potentially detect tissue damage caused by muzzle pressure on PMCT.

## Main text

### Materials and methods

#### Study material

Pig carcasses have been widely utilized as substitutes for human cadavers in forensic studies [11, 12].

In this study, a total of eight piglet carcasses were used as the study material. The carcasses (weight range 2–4 kg) were collected from a local farm, having died of natural causes within 24 h prior to our experiment. Carcasses with external deformities or skin abnormalities were excluded.

In accordance with the local regulations, ethical approvals were not required, as this study did not involve laboratory animals, living animals, human cadavers, or living human subjects. The piglets were raised as conventional livestock (not for research purposes) and they had died of natural causes prior to this study. The disposal of the carcasses was performed according to the local regulations immediately after data collection.

#### Infliction of gunshot wounds

We used the Ruger Standard Model semiautomatic pistol in caliber a .22 Long Rifle (5.6 × 15 mm), 51/2 barrel length version of Mark II model, with Lapua Pistol King cartridge with 2.59 g (40 grain) round nose unjacketed lead bullet. Reported muzzle velocity with the barrel length of 120 mm is 270 m/s. Firearm and bullet types were selected in accordance with the fact that .22 Long Rifle handguns are often used in homicides [13].

The carcasses were randomized to 5–10 gunshots depending on their size (average 7.4 shots per carcass) from the following distances: 0 cm (contact shot), 20 cm (close-range shot), or 100 cm (distant shot). The gunshots were fired vertically in a 90° angle to the left or right side of the carcass, excluding the head and the distal extremities. Accurate shooting distance was ensured with an adjustable ruler. We assumed that the shooting distance, varying from 0 to 100 cm, would not have a significant impact on bullet velocity and the wound caused by the bullet.

#### Post-mortem computed tomography

Carcasses were scanned using clinical CT equipment (Somatom Definition Flash, Siemens Healthcare, Forcheim, Germany), with a peak kilovoltage of 80, rotation time of 1 s, tube-current of 536 mA, pitch factor of 0.35, and collimation of 128 × 0.6. Images were reconstructed using I44s kernel with Safire setting 3, slice thickness of 0.6 mm, and slice interval of 0.6 mm. The reconstruction diameter was adjusted between 134 and 225 mm, ensuring that the entire torso of the carcass was within the display field of view. Kilovoltage and tube current were selected in order to optimize the contrast-to-noise ratio of images.

The PMCT scans were evaluated with OsiriX MD version 12.0.1 (Pixmeo SARL, Geneva, Switzerland) by the first author of the paper. Entrance wounds were first located using axial scans and then examined in appropriate planes, depending on wound location and bullet trajectory within tissues.

**Fig. 1:**
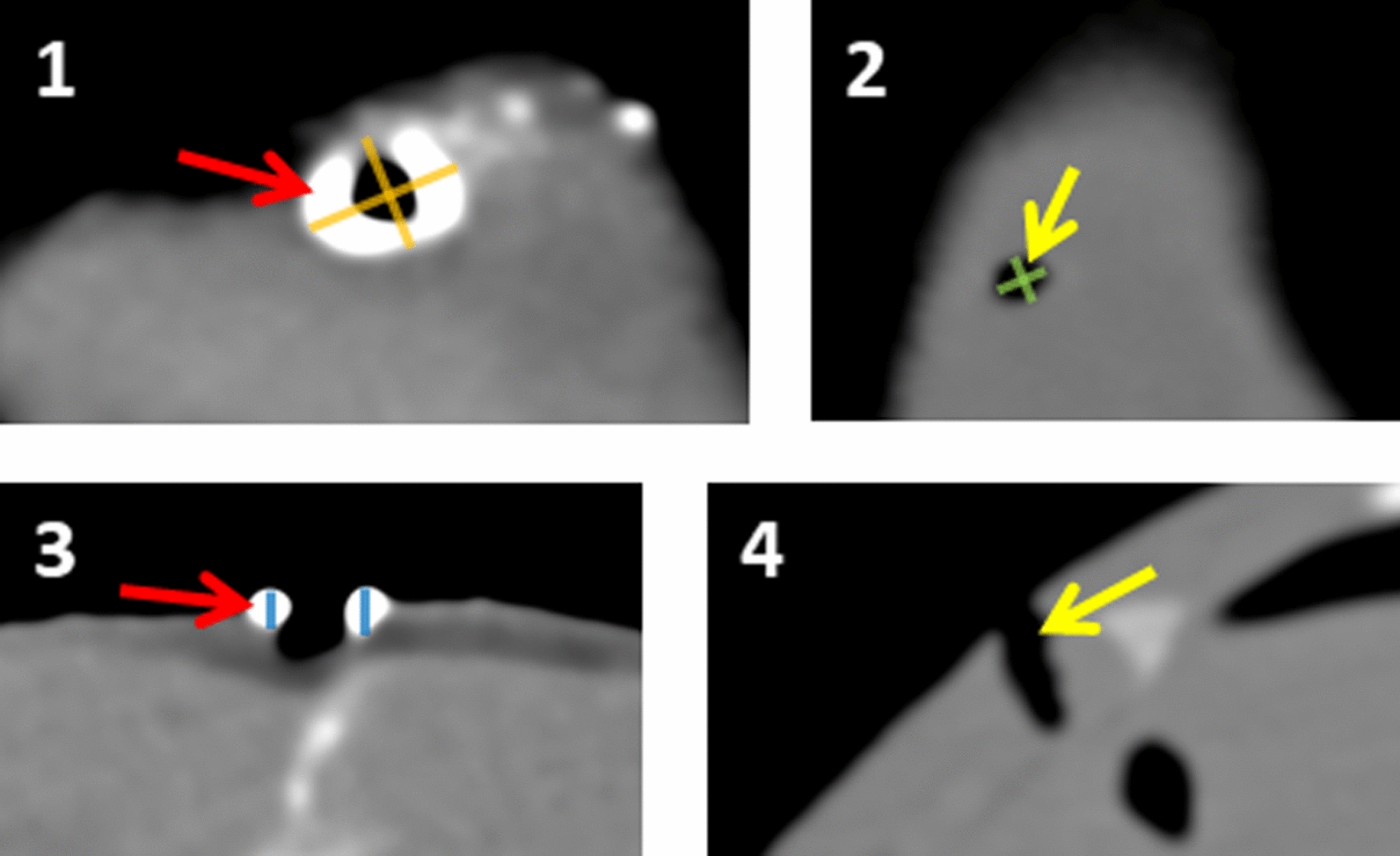
PMCT scan of a typical contact shot wound (1 = transverse view, 3 = longitudinal view) and a typical close-range/distant shot wound (2 = transverse view, 4 = longitudinal view). Note the hyperdense ring that is surrounding the outermost part of the contact shot wound channel (red arrows) but is absent from the close-range/distant shot wounds (yellow arrows). Three measurements were obtained from the scans (channel diameter = mean of green measurements; ring diameter = mean of orange measurements; ring thickness = mean of blue measurements); please find numerical values in Table 1 and Additional file 2: Table S2

Figure 1 illustrates the measurements obtained from the PMCT scans:

1. *Channel diameter*, i.e., the cross-sectional diameter of the outermost part of the wound channel, defined as the mean of two perpendicular measurements;
2. *Ring diameter*, i.e., the cross-sectional diameter of the area delineated by the high density ring surrounding the contact shot wound channel, defined as the mean of two perpendicular measurements; and
3. *Ring thickness*, i.e., the thickness of the high density ring surrounding the contact shot wound channel, defined as the mean of two measurements from the opposite sides of the ring.

The measurements were obtained using the measurement tool in OsiriX (accuracy of 0.1 mm). To evaluate intra-observer reliability (i.e., the degree of concordance in measurements taken twice by the same individual), a second measurement round comprising 10 randomly selected scans of each category was performed by the same researcher under identical conditions two weeks after the initial round.

#### Statistical analysis

IBM SPSS Statistics version 26 (IBM, Armonk, NY, USA) and Stata/MP version 16 (StataCorp, College Station, TX, USA) were used to perform the statistical analyses. P values < 0.05 were considered statistically significant. The characteristics of the PMCT measurements were presented as means and standard deviations (SDs). Intra-rater reliability of the PMCT variables was analyzed by comparing two separate but identical measurement rounds performed by one rater; intraclass correlation coefficient (ICC) was then calculated using the two-way mixed model with the absolute agreement type for single measures [14]. Differences between shooting distance categories were analyzed by multinomial logistic regression.

### Results

**Fig. 2:**
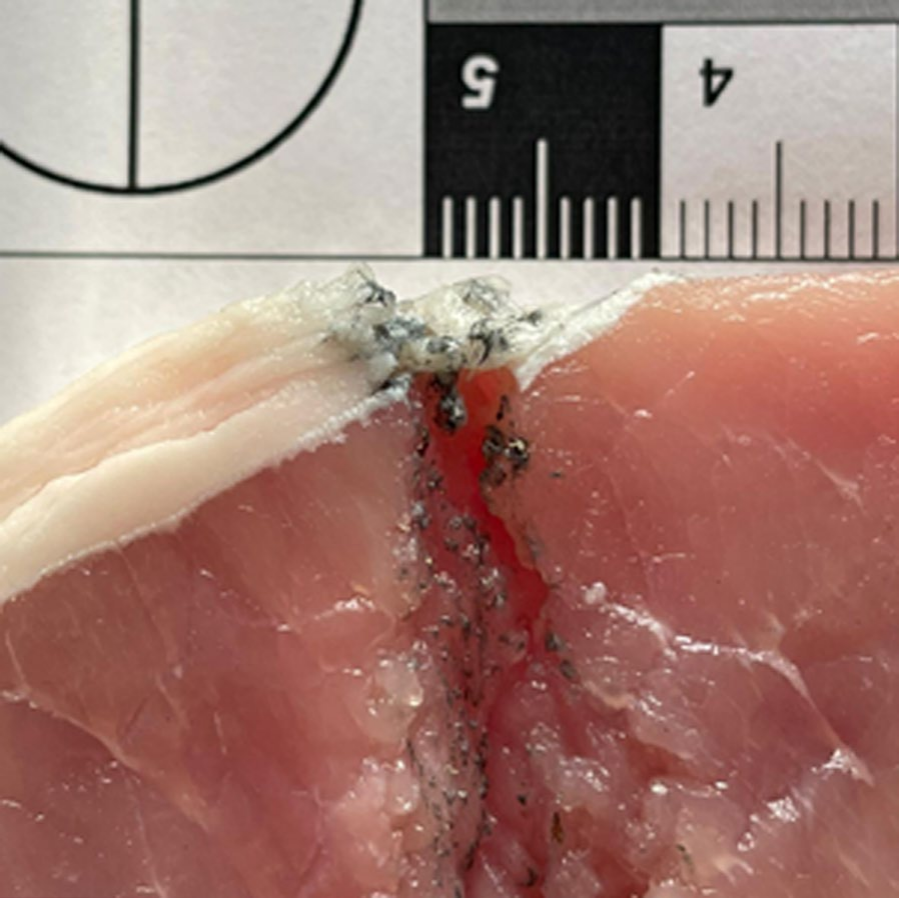
Macroscopic cross-section of a contact shot wound channel. Random deposits of gunshot residue can be seen in the wound channel, but a clear ring corresponding to PMCT findings is missing from the outer end

A total of 59 gunshot wounds were identified from the PMCT scans. Figure 1 shows the typical appearances of a contact shot wound and a close-range/distant shot wound. Notably, contact shot wounds had a hyperdense ring-shaped area surrounding the outermost part of the wound channel. The radiodensity of the ring could not be measured reliably as the ring demonstrated a great deal of variation in Hounsfield unit (HU) values. Gunshot residue appeared not to be a major component in this finding, as it was spread quite randomly inside the wound channel (Fig. 2). Close-range and distant shot wounds did not have this feature and they were not distinguishable from each other on PMCT.

One to three numerical measurements were taken from each wound, as demonstrated by Fig. 1. The intra-rater reliability of these measurements was good to excellent (ICC 0.73–0.94; Additional file 1: Table S1). The mean diameter of the wound channel ranged from 3.4 to 5.3 mm, being smallest in contact shots and largest in distant shots. Both close-range shots and distant shots showed a statistically significant difference to contact shot wounds (p ≤ 0.002; Table 1). In contact shot wounds, the mean diameter of the area delineated by the hyperdense ring was 7.8 mm, and the mean thickness of the ring was 2.4 mm (Table 1). Individual measurements are presented in Additional file 2: Table S2.

**Table 1:** Means and standard deviations of PMCT variables in gunshot distance groups, with P values against the contact shot group

Variable	Contact shot (n = 23)		Close-range shot (n = 21)			Distant shot (n = 15)		
	Mean	SD	Mean	SD	P value^1^	Mean	SD	P value^1^
Channel diameter (mm)	3.4	0.7	4.5	0.7	0.002	5.3	0.8	< 0.001
Ring diameter (mm)	7.8	1.3	Not visible			Not visible		
Ring thickness (mm)	2.4	0.6	Not visible			Not visible		

Individual measurements are presented in Additional file 2: Table S2

*SD* standard deviation

^1^P values against the contact shot group

### Discussion

This PMCT study revealed clear differences in wound characteristics between contact shots (0 cm), close-range shots (20 cm), and distant shots (100 cm). The most prominent observation in our study was the ring-shaped rim around the entrance wound of contact shots; it was not observed in close-range or distant wounds. Additionally, the diameter of the entrance wound increased with shooting distance, being smallest in contact shots and largest in distant shots.

Conventional methods to evaluate shooting distance [1–3] are not always applicable in forensic cases involving gunshot victims. This may be the case when, e.g., the victim’s clothes are disposed or skin is washed [15, 16]. Several factors such as clothing influence the estimation and measurement of gunshot residue deposits. We therefore hypothesized that a postmortem imaging approach might bring additional value to the estimation of shooting distance.

In the case of a firm contact shot, gases exit the barrel before the projectile, resulting in the laceration and tearing of superficial tissues. In a contact shot, most of the gunshot residue is also located inside the wound and may thus not be externally visible. According to previous studies, the nature of injuries inflicted by exit gases is largely dependent on the type of tissue underlying the wound; as for soft tissue, the subcutaneous damage should be relatively similar between shooting distances [4].

In our data, all contact shot wounds were characterized by an easily recognized hyperdense ring surrounding the wound channel. We believe that the ring consists of subcutaneous tissue and potentially also gunshot residue. It would seem unlikely that the ring was comprised of a so-called bullet wipe [17] as the phenomenon was only observed in contact shots. According to the increased HU values, it seems plausible that radio-opaque components in gunshot residue may play a role in the increased radiodensity. Importantly, the presence of a circular rim around the entrance wound on a PMCT scan could clearly contribute to the estimation of shooting distance, as it would confirm a potential contact shot in cases where the conventional signs of a contact shot are absent.

Interestingly, the diameter of the entrance wound channel increased with shooting distance. In contact shots, the wound channel was very narrow, and in distant shots, the wound channel was approximately of the same diameter as the actual bullet. While we could not confirm the mechanism behind this observation, we believe it is associated with the combination of increased hydrostatic shock and exhaust gases. The finding contradicted previous studies which have suggested that exhaust gases blown into the tissue can cause additional cavitation in contact and close-range shots [18]. However, while there are a number of studies aiming to infer circumstantial factors from wound channel size, the conventional methods mostly build upon bone defects instead of soft tissue, and the conclusions remain imprecise [19]. As for PMCT, previous studies have focused on gunshot residue instead of wound channel dimensions [10, 20–22]. Hence, the present study appears to be among the first to utilize PMCT in the association between shooting distance and the dimensions of the wound channel in soft tissue.

There were several strengths in our study. As we utilized low velocity, round nose, lead bullets, we minimized the potential effects of deformation and fragmentation of the bullet on the wound channel. As our shooting distances were 0 cm, 20 cm, and 100 cm, the bullet velocity remained virtually similar regardless of shooting distance. The PMCT scans were obtained using a clinical device which could be readily used for forensic imaging.

In conclusion, this study provided further means to examine and interpret gunshot wounds on PMCT. In particular, the presence of a circular rim around the entrance wound on a PMCT scan could contribute to the estimation of shooting distance, as it would confirm a potential contact shot in cases where the conventional signs of a contact shot are absent. Future studies are encouraged to confirm the present findings in other samples and other firearm and ammunition types.

### Limitations

As our material comprised piglet carcasses instead of human cadavers, further studies are needed to explore the applicability of this method in human samples. It should also be acknowledged that gunshot injuries may differ markedly between cadavers and living individuals. In addition, we could only utilize piglets that had died within 24 h prior to our experiment. We used only one firearm and bullet type with three discrete shooting distances due to the preliminary nature of the study.

## Supplementary Information

**Additional file 1: Table S1.** Intra-rater reliability of the PMCT variables (n = 10 repeated measurements each).

**Additional file 2: Table S2.** Individual PMCT measurements.

## Data Availability

The datasets analyzed during the study are available from the corresponding author on reasonable request.

## Abbreviations

CT: Computed tomography
HU: Hounsfield unit
ICC: Intraclass correlation coefficient
PMCT: Post-mortem computed tomography
SD: Standard deviation
