# Supplementary material for: Post-mortem computed tomography in forensic shooting distance estimation: a porcine cadaver study
Source: BMC Res Notes. 2022 Mar 16;15:103. doi: 10.1186/s13104-022-05997-2 (PMC8925149; doi:10.1186/s13104-022-05997-2)
Supplement: Supplementary file 1 — Additional file 1: Table S1. Intra-rater reliability of the PMCT variables (n = 10 repeated measurements each). [file 13104_2022_5997_MOESM1_ESM.docx]

**Additional file 1**

**Table S1**. Intra-rater reliability of the PMCT variables (n = 10 repeated measurements each).

| Variable | ICC | 95% CI |
| --- | --- | --- |
| Channel diameter (mm) | 0.81 | 0.39—0.95 |
| Ring diameter (mm) | 0.94 | 0.78—0.99 |
| Ring thickness (mm) | 0.73 | 0.21—0.93 |

CI = Confidence interval, ICC = Intraclass correlation coefficient
