# Supplementary material for: Post-mortem computed tomography in forensic shooting distance estimation: a porcine cadaver study
Source: BMC Res Notes. 2022 Mar 16;15:103. doi: 10.1186/s13104-022-05997-2 (PMC8925149; doi:10.1186/s13104-022-05997-2)
Supplement: Supplementary file 2 — Additional file 2: Table S2. Individual PMCT measurements. [file 13104_2022_5997_MOESM2_ESM.docx]

**Additional file 2**

**Table S2**. Individual PMCT measurements.

| Shot type | Channel diameter (mm) | Ring diameter (mm) | Ring thickness (mm) |
| --- | --- | --- | --- |
| Contact shot | 2.7 | 7.5 | 2.2 |
| Contact shot | 3.6 | 7.0 | 2.4 |
| Contact shot | 2.8 | 6.9 | 1.9 |
| Contact shot | 4.0 | 8.3 | 2.2 |
| Contact shot | 4.3 | 9.0 | 2.0 |
| Contact shot | 4.1 | 7.3 | 2.3 |
| Contact shot | 3.0 | 9.4 | 2.6 |
| Contact shot | 3.6 | 9.1 | 2.4 |
| Contact shot | 3.2 | 7.5 | 2.6 |
| Contact shot | 4.1 | 7.5 | 2.7 |
| Contact shot | 3.0 | 5.7 | 1.9 |
| Contact shot | 4.2 | 8.8 | 2.9 |
| Contact shot | 2.5 | 5.1 | 2.4 |
| Contact shot | 3.6 | 6.7 | 1.8 |
| Contact shot | 2.6 | 8.0 | 2.5 |
| Contact shot | 2.9 | 8.1 | 2.0 |
| Contact shot | 3.3 | 7.5 | 2.1 |
| Contact shot | 3.7 | 7.3 | 2.0 |
| Contact shot | 4.3 | 10.1 | 2.1 |
| Contact shot | 2.4 | 8.3 | 1.8 |
| Contact shot | 3.9 | 8.3 | 2.3 |
| Contact shot | 4.9 | 10.1 | 2.9 |
| Close-range shot | 2.6 | 6.6 | 4.4 |
| Close-range shot | 3.9 | n/a | n/a |
| Close-range shot | 6.3 | n/a | n/a |
| Close-range shot | 4.1 | n/a | n/a |
| Close-range shot | 4.5 | n/a | n/a |
| Close-range shot | 4.8 | n/a | n/a |
| Close-range shot | 4.9 | n/a | n/a |
| Close-range shot | 3.8 | n/a | n/a |
| Close-range shot | 5.0 | n/a | n/a |
| Close-range shot | 4.8 | n/a | n/a |
| Close-range shot | 4.0 | n/a | n/a |
| Close-range shot | 4.6 | n/a | n/a |
| Close-range shot | 4.2 | n/a | n/a |
| Close-range shot | 4.1 | n/a | n/a |
| Close-range shot | 4.3 | n/a | n/a |
| Close-range shot | 5.0 | n/a | n/a |
| Close-range shot | 5.6 | n/a | n/a |
| Close-range shot | 4.9 | n/a | n/a |
| Close-range shot | 3.6 | n/a | n/a |
| Close-range shot | 3.8 | n/a | n/a |
| Close-range shot | 3.9 | n/a | n/a |
| Distant shot | 3.7 | n/a | n/a |
| Distant shot | 6.8 | n/a | n/a |
| Distant shot | 5.6 | n/a | n/a |
| Distant shot | 4.8 | n/a | n/a |
| Distant shot | 4.6 | n/a | n/a |
| Distant shot | 5.7 | n/a | n/a |
| Distant shot | 4.4 | n/a | n/a |
| Distant shot | 5.7 | n/a | n/a |
| Distant shot | 4.1 | n/a | n/a |
| Distant shot | 5.6 | n/a | n/a |
| Distant shot | 5.7 | n/a | n/a |
| Distant shot | 5.3 | n/a | n/a |
| Distant shot | 6.0 | n/a | n/a |
| Distant shot | 5.2 | n/a | n/a |
| Distant shot | 6.5 | n/a | n/a |

n/a = Not applicable
